# Impact of geriatric impairments on outcomes of single‐agent immunotherapy in solid tumors

**DOI:** 10.1002/ijc.70185

**Published:** 2025-10-04

**Authors:** Asli Özkan, Karlijn de Joode, Ellen Kapiteijn, Marije Slingerland, Stephanie Zunder, Frederiek van den Bos, Simon Mooijaart, Anna Uit den Boogaard, Stella Trompet, Hans Westgeest, Astrid van der Veldt, Ron H. J. Mathijssen, Geert Labots, Cynthia Holterhues, Els M. E. Verdegaal, Nienke A. de Glas, Johanneke E. A. Portielje

**Affiliations:** ^1^ Department of Medical Oncology Leiden University Medical Center Leiden The Netherlands; ^2^ Department of Medical Oncology Erasmus University Medical Center Rotterdam The Netherlands; ^3^ Department of Internal Medicine, Section of Gerontology and Geriatrics Leiden University Medical Center Leiden The Netherlands; ^4^ LUMC Center for Medicine for Older People Leiden University Medical Center Leiden The Netherlands; ^5^ Department of Medical Oncology Amphia Hospital Breda The Netherlands; ^6^ Department of Radiology & Nuclear Medicine Erasmus University Medical Center Rotterdam The Netherlands; ^7^ Department of Internal Medicine Haga Hospital The Hague The Netherlands; ^8^ Department of Medical Oncology, Oncode Institute Leiden University Medical Center Leiden The Netherlands; ^9^ Department of Medical Oncology Helse Førde Førde Norway

**Keywords:** frailty, geriatric assessment, geriatric screening, immune checkpoint inhibitors, immune‐related adverse events

## Abstract

Cancer is increasingly prevalent among older adults with geriatric impairments, yet the impact of frailty on immune checkpoint inhibitor (ICI) therapy outcomes remains underexplored. This study aims to assess the association between frailty and grade ≥3 immune‐related adverse events (irAEs), clinical benefit, all‐cause hospitalization, and mortality in older patients undergoing ICI therapy. Patients aged ≥65 years, treated with anti‐PD‐1 monotherapy for a solid malignancy (September 2018–February 2024), were prospectively included in this multicenter study. The association between frailty, components of the geriatric assessment, and number of impaired geriatric domains with the occurrence of grade ≥3 irAEs, all‐cause hospitalization, and clinical benefit was analyzed using univariable and multivariable logistic regression. Cox proportional hazards models were used to analyze all‐cause mortality. Among the 110 patients, 55% were classified as frail. Grade ≥3 irAEs occurred in 17.3%, with no significant difference between frail and non‐frail patients (18.0% vs. 16.4%, *p* = .814). Frailty was associated with higher hospitalization (OR: 3.98, 95%C.I.: 1.20–13.19) and mortality risk (HR: 5.23, 95%C.I.: 1.81–15.11). Multimorbidity (Charlson comorbidity index score ≥3) was also associated with hospitalization (OR: 5.54, 95%C.I.: 1.81–16.99). Frailty and the number of impaired geriatric domains were not associated with clinical benefit of ICIs in the palliative treatment setting (*p* = .374, and *p* = .155, respectively). Frailty should not be considered a contraindication for ICI therapy, as this therapy is generally well‐tolerated, even among older frail patients. Instead, frailty should be viewed as a relevant clinical factor for optimizing therapeutic decision‐making and tailoring supportive interventions in older patients.

## What's New?

This study comprehensively assesses the impact of frailty and geriatric impairments on key clinical outcomes of single‐agent immunotherapy in older adults with solid tumors. Using prospective multicenter data, the authors demonstrate that frailty is significantly associated with higher rates of all‐cause hospitalization and mortality. However, frailty does not predict severe immune‐related toxicity, or lack of treatment response in the palliative care setting. These findings highlight the safety and tolerability of immune checkpoint inhibitors in frail older patients and support the inclusion of geriatric screening in risk stratification to optimize oncology care in aging populations.

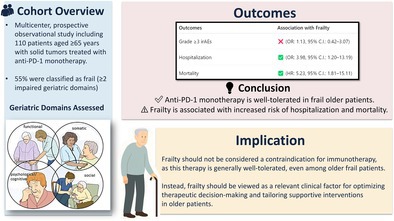

**Abbreviations**
6‐CIT: Six‐Item Cognitive Impairment Test
ADL: activities of daily living
AE: adverse event
CCI: Charlson comorbidity index
CT: computed tomography
CTCAE: common terminology criteria for adverse events
ECOG: Eastern Cooperative Oncology Group
EQ‐5D‐3L: EuroQol 5‐dimension 3‐level
G8: geriatric 8 screening tool
GA: geriatric assessment
HR: hazard ratio
IADL: instrumental activities of daily living
ICI: immune checkpoint inhibitor
IMAGINE: immunotherapy in AGINg patiEnts study
IQR: interquartile range
irAE: immune‐related adverse event
MNA‐SF: mini nutritional assessment—short form
MRI: magnetic resonance imaging
OR: odds ratio
PD‐1: programmed death‐1
PET‐CT: positron emission tomography‐computed tomography
PHQ‐2: patient health questionnaire‐2
RECIST: response evaluation criteria in solid tumors
SPSS: statistical package for the social sciences
TENT: triage of elderly needing treatment study

## 1 INTRODUCTION

The global population is aging, leading to a significant rise in cancer incidence among older adults.^1^ Older adults with cancer comprise a heterogeneous population, often presenting with geriatric impairments, including functional decline, multiple comorbidities, and polypharmacy, making them more vulnerable to the adverse effects of oncological treatments. While chronological age and Eastern Cooperative Oncology Group (ECOG) performance status are commonly used in treatment decision‐making, they do not fully capture a patient's vulnerability. Frailty has been shown to be a strong predictor of treatment outcomes in older adults with cancer; however, research indicates that it is often underrecognized in clinical practice. This highlights the need for objective assessment.^2^

Previous studies have shown that geriatric impairments identified through geriatric assessment (GA) predict chemotherapy toxicity, and that targeted interventions and dose adjustments can reduce serious toxic effects.^3^, ^4^, ^5^, ^6^ These findings underscore the importance of geriatric evaluations in cancer treatment. GA is a comprehensive assessment using validated scales to identify impairments in the four geriatric domains: somatic, functional, mental (encompassing both psychological and cognitive functioning), and social. However, the role of GA and screening in predicting adverse outcomes in patients undergoing immunotherapy remains underexplored.^3^, ^7^

Immune checkpoint inhibitors (ICIs) have revolutionized cancer treatment for several indications, improving survival rates.^8^, ^9^ However, ICIs can cause immune‐related adverse events (irAEs), ranging from mild to severe, potentially leading to significant organ function decline and impacting quality of life.^10^, ^11^ ICIs generally have a safer toxicity profile than chemotherapy, and age is not a predictor for irAEs.^12^, ^13^, ^14^ Similarly, previous studies showed that age does not predict ICI efficacy.^15^, ^16^, ^17^ However, these studies did not account for frailty, a common condition among older adults, as a potential predictor of response. The role of frailty in influencing irAEs and ICI efficacy remains largely unexplored in clinical oncology.^7^, ^18^ This is partly because pivotal trials excluded patients with poor ECOG performance status. Although ECOG performance status does not fully capture frailty, many frail older cancer patients have a poor ECOG score, leading to their underrepresentation in clinical trials.^7^, ^19^

Geriatric impairments may increase the risk of grade ≥3 irAEs in older patients.^7^ Management of grade ≥3 irAEs often involves discontinuing ICI therapy and using corticosteroids or other immunosuppressants, which can induce significant side effects when used for a longer time in older patients, including diabetes mellitus and opportunistic infections.^20^ Age‐related physiological changes and pre‐existing comorbidities can further complicate the management of irAEs. This underscores the critical need to understand the interplay between geriatric impairments and irAEs to optimize treatment strategies and improve patient care. Hence, the primary objective of this study is to assess the association between geriatric impairments and the occurrence of grade ≥3 irAEs, clinical benefit, all‐cause hospitalization, and mortality in older patients undergoing single‐agent immunotherapy.

## 2 MATERIALS AND METHODS

### 2.1 Patient selection

Patients in this study were selected from two multicenter, prospective, observational studies: the Triage of Elderly Needing Treatment (TENT) study and Immunotherapy in AGINg patiEnts (IMAGINE) study.

The TENT study included patients aged 70 and older who required medical interventions or for whom treatment was an option (e.g., immunotherapy). It utilized a short geriatric screening to assess clinical status, with those scoring abnormally receiving a comprehensive geriatric assessment. The IMAGINE study included patients aged 65 years and older with a solid malignancy, at any stage, who were scheduled for treatment with ICIs. Baseline geriatric screening was conducted using multiple screening tools. For more detailed information, refer to Section 2.2. Both studies aimed to predict clinical outcomes (e.g., quality of life, treatment toxicity, clinical benefit) in older patients undergoing cancer treatment (see Section 2.3). Detailed information on the data registry of both studies has been previously published.^21^

The inclusion criteria for this current study required that patients must be 65 years or older and must have started single‐agent ICI therapy for a solid malignancy. Patients receiving concomitant therapies, such as chemotherapy or dual immunotherapy, were excluded from the study. We included only patients with a minimum available follow‐up time of 6 months, allowing sufficient time for the development of irAEs.

All included patients were treated at one of the following hospitals: Leiden University Medical Center (Leiden, the Netherlands), Erasmus University Medical Center (Rotterdam, the Netherlands), Amphia Hospital (Breda, the Netherlands), or Haga Hospital (Den Haag, the Netherlands).

### 2.2 Baseline characteristics and geriatric screening

Baseline characteristics included age, sex, type of cancer, treatment intent, immunotherapy regimen, and ECOG performance status. In both studies, frailty was assessed across four geriatric domains—somatic, functional, mental, and social—using validated screening tools either in person or by telephone before treatment initiation, or within 2 weeks after the start of therapy. This approach followed a similar methodology to that of a previous study.^22^ In the TENT study, geriatric screening consisted of the Geriatric 8 (G8) questionnaire and the Six‐Item Cognitive Impairment Test (6‐CIT).^23^ A comprehensive GA, including physical capacity tests, was performed only if either the G8 score was ≤14 or the 6‐CIT suggested cognitive impairment.^23^ In contrast, the IMAGINE study conducted a GA in all patients aged ≥65 years. The design and methodology of this study have been described previously.^21^ Within this GA, the somatic domain included an evaluation of baseline comorbidity using the Charlson comorbidity index (CCI), polypharmacy, and nutritional status, which was assessed using the Mini Nutritional Assessment—Short Form (MNA‐SF), supplemented by the G8 questionnaire if the MNA‐SF was missing.^24^, ^25^ In cases where comorbidity information could not be obtained from the medical history, data was extracted from digital patient files, and the CCI was calculated based on this information. The functional domain was assessed through the Katz Index of Independence in activities of daily living (ADL), alongside inquiry about falls within the past 6 months prior to therapy initiation, and the Lawton instrumental activities of daily living (IADL) to assess independence.^26^, ^27^, ^28^ The mental domain included screening for cognitive impairment or dementia using the 6‐CIT, and symptoms of depression using the patient health questionnaire‐2 (PHQ‐2).^29^, ^30^ The social domain was assessed by inquiring about the current living situation. Additionally, the EuroQoL 5‐dimension 3‐level (EQ‐5D‐3L) index score was used to assess quality of life.^31^, ^32^

Similar to a previous publication, participants were classified as frail if they exhibited impairment in two or more of the four geriatric domains.^22^ A domain was considered abnormal if one or more tests within it yielded an abnormal score.^22^ The operational definition of frailty was consistent across both cohorts. The thresholds for geriatric impairment within assessment/screening tools across the four domains are presented in Supplementary Table 1.

### 2.3 Follow‐up data

Follow‐up data, including occurrence of grade ≥3 irAEs, treatment response to ICI, first all‐cause unplanned hospital admission and mortality status, was extracted from patient charts as part of the study protocol. Grade ≥3 irAEs were defined according to the Common Terminology Criteria for Adverse Events (CTCAE) Version 5.0.^33^ Clinicians collected adverse event data from patient records, ensuring data reliability. Treatment response was evaluated at 3 and 6 months (or earlier if clinical progression was suspected) as per standard of care, using CT, PET‐CT and/or MRI imaging. Response was mostly evaluated according to the Response Evaluation Criteria in Solid Tumors (RECIST) version 1.1. For the current study, radiological response at 3 months was categorized as complete (including disease‐free), partial, stable, mixed, or progressive. Patients with a complete or partial response were classified as having clinical benefit. In case of stable disease or mixed response at 3 months, further assessment was deferred until the 6‐month follow‐up. At 6 months, complete or partial response continued to be categorized as a clinical benefit, and stable disease at 6 months was also classified as a clinical benefit. Patients exhibiting progression at either 3 or 6 months were classified as having no clinical benefit. Follow‐up in the TENT study continued for 12 months and up to 24 months in the IMAGINE study, or until death. Mortality data were obtained from digital patient records or municipal registries.

### 2.4 Statistical analyses

The chi‐square test and Mann–Whitney tests were used to assess baseline differences between frail and non‐frail patients who participated in the TENT and IMAGINE studies. All analyses were performed using SPSS version 29 (IBM Corp), with significance set at *p* <.05.

Univariable and multivariable logistic regression analyses were performed to assess the association between frailty, impaired components of the GA, the number of impaired geriatric domains (categorized as 0–1, 2, or 3–4), and the occurrence of grade ≥3 irAEs, including the calculation of odds ratios (ORs) and 95% confidence intervals (CIs). The GA components were treated as independent variables, with the presence of grade ≥3 irAEs as the dependent variable. Adjustments were made for potential confounding factors, including age, sex, treatment intent, and tumor type. Univariable and multivariable logistic regression was used to investigate the predictive value of frailty, impaired components of the GA, and the number of impaired geriatric domains for hospital admission. Cox proportional hazards models were used to evaluate the impact of frailty, number of impaired geriatric domains, and baseline GA components on mortality rates, with adjustments for the same covariates mentioned above.

## 3 RESULTS

### 3.1 Patient characteristics

Between September 2018 and February 2024, the IMAGINE study included 84 patients and the TENT study included 26 patients who underwent anti‐PD‐1 therapy for a solid tumor. Median follow‐up time was 12.4 months (IQR 7.3–14.7).

Patient characteristics are described in Table 1A. Among all patients, 61 (55%) were classified as frail. The median age was similar between the two groups (*p* = .523). Tumor type distribution differed significantly between the two groups (*p* = .042). Melanoma was the most common tumor type overall (73%). Patients with frailty had more often lung cancer (23% vs. 8%, *p* = .042). Frail patients were more frequently treated in a palliative setting (85% vs. 55%, *p* = .002). The ECOG performance status was comparable between the two groups (*p* = .217).

**TABLE 1A:** Baseline patient characteristics.

	Participant, No. (%)			
Variable	Total (*N* = 110)	Without frailty^a^ (*N* = 49)	With frailty^a^ (*N* = 61)	*p*‐value
Sex	‐	‐	‐	
Male	75 (68)	38 (78)	37 (61)	.*059*
Female	35 (32)	11 (22)	24 (39)	
Age (median, IQR), year	73 (70–77)	72 (70–77)	73 (70–80)	.*523*
Type of cancer	‐	‐	‐	
Melanoma	80 (73)	42 (86)	38 (62)	*.042*
Lung	18 (16)	4 (8)	14 (23)	
Genitourinary	5 (5)	0 (0)	5 (8)	
Head and neck	4 (4)	2 (4)	2 (3)	
Colon	3 (3)	1 (2)	2 (3)	
Treatment intent	‐	‐	‐	
Curative	28 (25)	19 (39)	9 (15)	.*002*
Palliative	79 (72)	27 (55)	52 (85)	
Unknown	3 (3)	3 (6)	0 (0)	
ECOG performance status^b^	‐	‐	‐	
0	50 (45)	27 (55)	23 (38)	.*217*
1	37 (34)	14 (29)	23 (38)	
≥2	6 (5)	1 (2)	5 (8)	
Unknown	17 (15)	7 (14)	10 (16)	

^a^ Frailty was defined as an impairment in ≥2 of 4 geriatric domains.

^b^ The Eastern Cooperative Oncology Group Performance Status is a scale that indicates a cancer patient's general condition. Numbers range from 0 to 5, with higher numbers representing a poorer general condition.

Geriatric characteristics were evaluated across several domains at baseline, and the distribution of the sum of impaired domains among the patients is presented in Table 1B. Overall, 50% of the cohort had an impaired G8 score (≤14). A higher proportion of patients without frailty had a normal G8 score (65%) compared to those with frailty (38%). Notably, frail patients had more comorbidities (26 vs. 14%). Polypharmacy (≥5 medications) was more prevalent among frail patients (52% vs. 27%). Additionally, frail patients were more likely to be at risk of malnutrition (64% vs. 33%). In terms of functional status, frail patients had a higher incidence of falls (31% vs. 6%) in the 6 months prior to ICI treatment and were significantly more dependent on iADLs (39% vs. 2%). Cognitive impairment was present in 23% of frail patients, whereas all non‐frail patients had normal cognition scores. Living alone was also more common among frail patients (33%) compared to non‐frail patients (8%).

**TABLE 1B:** Baseline geriatric characteristics.

	Participants, No. (%)			
		Total (*N* = 110)	Without frailty^a^ (*N* = 49)	With frailty^a^ (*N* = 61)
Sum of impaired domains	0	10	10	0
	1	39	39	0
	2	36	0	36
	3	20	0	20
	4	5	0	5
Baseline geriatric characteristics measured				
Screening of all 4 domains	G8 questionnaire^c^	‐	‐	‐
	Normal	55 (50)	32 (65)	23 (38)
	Impaired	55 (50)	17 (35)	38 (62)
Somatic	CCI comorbidity^d^	‐	‐	‐
	0	35 (32)	23 (47)	12 (20)
	1	31 (28)	10 (20)	21 (34)
	2	21 (19)	9 (18)	12 (20)
	≥3	23 (21)	7 (14)	16 (26)
	Polypharmacy^e^	‐	‐	‐
	No	65 (59)	36 (73)	29 (48)
	Yes	45 (41)	13 (27)	32 (52)
	MNA‐SF^f^	‐	‐	‐
	Well nourished	55 (50)	33 (67)	22 (36)
	Risk of malnutrition	55 (50)	16 (33)	39 (64)
Functional	Falls in last 6 months	‐	‐	‐
	No	88 (80)	46 (94)	42 (69)
	Yes	22 (20)	3 (6)	19 (31)
	ADL^g^	‐	‐	‐
	Independent	107 (97)	48 (98)	59 (97)
	Dependent	3 (3)	1 (2)	2 (3)
	IADL^h^	‐	‐	‐
	Independent	85 (77)	48 (98)	37 (61)
	Dependent	25 (23)	1 (2)	24 (39)
Cognitive^b^	6‐CIT^i^	‐	‐	‐
	Normal	94 (85)	49 (100)	45 (74)
	Impaired	14 (13)	0 (0)	14 (23)
	Unknown	2 (2)	0 (0)	2 (3)
Psychological^b^	PHQ‐2^j^	‐	‐	‐
	Normal	100 (91)	48 (98)	52 (85)
	Abnormal	10 (9)	1 (2)	9 (15)
Social	Living alone	‐	‐	‐
	No	86 (78)	45 (92)	41 (67)
	Yes	24 (22)	4 (8)	20 (33)

Abbreviations: 6‐CIT, Six‐Item Cognitive Impairment Test; ADL, activities of daily living; CCI, Charlson comorbidity index; G8, geriatric 8; IADL, Instrumental Activities of Daily Living; MNA‐SF, Mini Nutritional Assessment—Short Form; PHQ‐2, patient health questionnaire‐2.

^a^ Frailty was defined as an impairment in ≥2 of 4 geriatric domains.

^b^ Psychological and cognitive components fall under the same domain.

^**c**^ Defined as abnormal when G8 score ≤14.

^d^ Defined as CCI ≥1.

^**e**^ Defined as 5 or more medicines.

^f^ At risk of malnutrition when the score is ≤11.

^g^ Defined as dependent when ≤2.

^h^ Defined as dependent when ≤4 for men and ≤7 for women.

^i^ Defined as abnormal when >7.

^j^ Defined as abnormal when ≥3.

### 3.2 Grade ≥3 irAEs

Among the total cohort of 110 patients receiving anti‐PD‐1 immunotherapy, 19 patients (17.3%) developed grade ≥3 irAEs. Within the frail group, 11 out of 61 patients (18.0%) developed grade ≥3 irAEs, compared to 8 out of 49 patients (16.3%) in the non‐frail group (*p* = .814). Detailed information regarding the incidence and types of grade ≥3 irAEs is provided in Supplementary Table 2.

Of the 61 frail patients, 33 (54.1%) discontinued treatment prematurely. Among these, 12 (36.4%) discontinued due to (all grade) irAEs, 17 patients (51.5%) due to disease progression, and 4 patients (12.1%) due to poor overall condition. In contrast, 14 of the 49 non‐frail patients (28.6%) discontinued treatment prematurely, 8 (57.1%) due to irAEs, 6 (42.9%) discontinued treatment due to disease progression, and none (0%) due to poor overall condition. An overview of reasons for early treatment discontinuation of ICI for frail and non‐frail patients is provided in Supplementary Table 3.

Among the 27 hospitalized patients, 4 of the 21 frail patients (19.0%) and 2 of the 6 non‐frail patients (33.3%) were hospitalized specifically due to grade ≥3 irAEs.

### 3.3 Association of frailty, impaired GA components, and the number of impaired domains with grade ≥3 irAEs

Frailty was not significantly associated with the occurrence of grade ≥3 irAEs in both the univariable and multivariable analysis (OR: 1.52, 95%C.I.: 0.46–5.08, *p* = .495) (Table 2). The association between the number of impaired geriatric domains and the occurrence of grade ≥3 irAEs was also assessed in a multivariable analysis, using patients with 0–1 impaired domains as the reference group. There was no significant association for patients with 2 impaired domains (OR: 1.75, 95%C.I.: 0.51–6.06, *p* = .375). Similarly, no significant association was observed for patients with 3 or 4 impaired domains (OR: 0.80, 95%C.I.: 0.11–5.99, *p* = .828).

**TABLE 2:** Association between frailty, impairment in baseline geriatric characteristics, and grade ≥3 irAEs in older patients treated with anti‐PD‐1 immunotherapy.

	% of grade ≥3 irAEs within subgroup	Univariable logistic regression Odds ratio (95%C.I.)	*p*‐value	Multivariable logistic regression^a^ Odds ratio (95%C.I.)	*p*‐value
Frailty^b^	‐	‐	‐		‐
No	*16.3*%	Ref.	.*814*	Ref.	.*495*
Yes	*18.0*%	1.13 (0.42–3.07)		1.52 (0.46–5.08)	
Baseline geriatric characteristics					
G8 questionnaire^c^	‐	‐	‐	‐	‐
Normal	*18.2*%	Ref.	.*801*	Ref.	.*606*
Impaired	*16.4*%	0.88 (0.33–2.37)		1.35 (0.43–4.23)	
CCI comorbidity^d^	‐	‐	‐	‐	‐
0	*20.0*%	Ref.	.*579*	Ref.	.*607*
1	*9.7*%	0.43 (0.10–1.83)		0.51 (0.11–2.42)	
2	*23.8*%	1.25 (0.34–4.59)		1.49 (0.36–6.16)	
≥3	*17.4*%	0.84 (0.22–3.28)		1.28 (0.27–6.14)	
Polypharmacy^e^	‐	‐	‐	‐	‐
No	*15.4*%	Ref.	.*530*	Ref.	.*383*
Yes	*20.0*%	1.38 (0.51–3.71)		1.65 (0.54–5.08)	
MNA‐SF^f^	‐	‐	‐	‐	‐
Well nourished	*16.4*%	Ref.	.*801*	Ref.	.*376*
Risk of malnutrition	*18.2*%	1.14 (0.42–3.06)		1.69 (0.53–5.37)	
Falls in last 6 months	‐	‐	‐	‐	‐
No	*15.9*%	Ref.	.*452*	Ref.	.*506*
Yes	*22.7*%	1.56 (0.49–4.91)		1.51 (0.45–5.15)	
ADL^g^	‐	‐	‐	‐	‐
Independent	*17.8*%	Ref.	*NA* ^k^	Ref.	*NA* ^k^
Dependent	*0*%	NA^k^		NA^k^	
IADL^h^	‐	‐	‐	‐	‐
Independent	*21.2*%	Ref.	.*077*	Ref.	.*165*
Dependent	*4.0*%	0.16 (0.02–1.23)		0.22 (0.03–1.87)	
6‐CIT^i^	‐	‐	‐	‐	‐
Normal	*16.0*%	Ref.	.*471*	Ref.	.*632*
Impaired	*21.4*%	1.44 (0.36–5.77)		1.68 (0.27–10.62)	
Unknown	*50.0*%	5.27 (0.31–88.91)		3.55 (0.18–72.0)	
PHQ‐2^j^	‐	‐	‐	‐	‐
Normal	*19.0*%	Ref.	*NA* ^k^	Ref.	*NA* ^k^
Abnormal	*0.0*%	NA^k^		NA^k^	
Living alone	‐	‐	‐	‐	‐
No	*17.4*%	Ref.	.*929*	Ref.	.*950*
Yes	*16.7*%	0.95 (0.28–3.17)		0.96 (0.24–3.80)	

Abbreviations: 6‐CIT, Six‐Item Cognitive Impairment Test; ADL, activities of daily living; CCI, Charlson comorbidity index; G8, geriatric 8; IADL, Instrumental Activities of Daily Living; MNA‐SF, Mini Nutritional Assessment—Short Form; PHQ‐2, patient health questionnaire‐2.

^a^ Multivariable logistic regression models were adjusted for age, sex, treatment intent, and tumor type. Sex, treatment intent, and tumor type were added to the model as binary variables and age was added as a continuous variable.

^b^ Frailty was defined as an impairment in ≥2 of 4 geriatric domains.

^c^ Defined as abnormal when G8 score ≤ 14.

^d^ Defined as CCI ≥1.

^e^ Defined as 5 or more medicines.

^f^ At risk of malnutrition when the score is ≤11.

^g^ Defined as dependent when ≤2.

^h^ Defined as dependent when ≤4 for men and ≤7 for women.

^i^ Defined as abnormal when >7.

^j^ Defined as abnormal when ≥3.

^k^ No analysis was performed as there were no events recorded in the specific subgroup.

Additionally, none of the other baseline GA components showed a significant association with the occurrence of grade ≥3 irAEs (Table 2).

### 3.4 Predictive value of frailty, impaired GA components, and number of impaired domains for hospital admission

Hospitalization occurred in 27 patients, of whom 21 (77.8%) were frail. An overview of hospitalization causes by frailty status is provided in Supplementary Table 4. Reasons for hospitalization were mostly unrelated to ICI treatment in both frail and non‐frail patients (81.0% and 66.7%, respectively).

The association of frailty, impaired GA components, and the number of impaired domains with hospital admission is summarized in Table 3. Frailty was significantly associated with hospital admission (OR: 3.98, 95%C.I.: 1.20–13.19, *p* = .024), but this was often caused by other factors than immunotherapy‐related adverse events (Supplementary Table 4). Supplementary Figure 1a illustrates hospital admission rates in palliative patients, stratified by frailty status, and shows that frail patients within this subgroup had a higher frequency of hospitalizations, though this difference was not statistically significant.

**TABLE 3:** Predictive value of frailty, impaired geriatric domains and baseline GA components for hospital admission.

	% of hospital admission within subgroup	Univariable logistic regression Odds ratio (95%C.I.)	*p*‐value	Multivariable logistic regression^a^ odds ratio (95%C.I.)	*p*‐value
Frailty^b^	‐	‐	‐	‐	‐
No	14.3%	Ref.	‐	Ref.	‐
Yes	32.8%	2.93 (1.12–7.66)	.*029*	3.98 (1.20–13.19)	.*024*
Impaired geriatric domains	‐	‐	‐	‐	‐
0–1 (not frail)	14.3%	Ref.	‐	Ref.	‐
2 (frail)	22.2%	1.71 (0.56–5.26)	.*346*	2.62 (0.71–9.72)	.*149*
3–4 (very frail)	48.0%	5.54 (1.81–16.99)	.*003*	8.85 (1.91–41.00)	.*005*
Baseline GA components	‐				
G8 questionnaire^c^	‐	‐	‐	‐	‐
Normal	18.2%	Ref.	‐	Ref.	‐
Impaired	30.9%	2.01 (0.83–4.92)	.*124*	1.13 (0.37–3.49)	.*834*
CCI comorbidity^d^	‐	‐	‐	‐	‐
0	17.1%	Ref.	‐	Ref.	‐
1	19.4%	1.16 (0.33–4.06)	.*816*	1.01 (0.24–4.17)	.*992*
2	19.0%	1.14 (0.28–4.61)	.*857*	0.70 (0.14–3.54)	.*670*
≥3	47.8%	4.43 (1.33–14.72)	.*015*	2.80 (0.62–12.76)	.*183*
Polypharmacy^e^	‐	‐	‐	‐	‐
No	23.1%	Ref.	‐	Ref.	‐
Yes	26.7%	1.21 (0.50–2.91)	.*667*	0.62 (0.18–2.10)	.*441*
MNA‐SF^f^	‐	‐	‐	‐	‐
Well nourished	20.0%	Ref.	‐	Ref.	‐
Risk of malnutrition	29.1%	1.64 (0.68–3.96)	.*270*	1.38 (0.49–3.94)	.*544*
Falls in last 6 Months	‐	‐	‐	‐	‐
No	21.6%	Ref.	‐	Ref.	‐
Yes	36.4%	2.08 (0.76–5.68)	.*155*	2.01 (0.63–6.45)	.*239*
ADL^g^	‐	‐	‐	‐	‐
Independent	23.4%	Ref.	‐	Ref.	‐
Dependent	66.7%	6.56 (0.57–75.40)	.*131*	5.34 (0.31–93.64)	.252
IADL^h^	‐	‐	‐	‐	‐
Independent	20.0%	Ref.	‐	Ref.	‐
Dependent	40.0%	2.67 (1.02–6.97)	.*045*	2.87 (0.92–8.96)	.*069*
6‐CIT^i^	‐	‐	‐	‐	‐
Normal	22.3%	Ref.	‐	Ref.	‐
Impaired	35.7%	1.93 (0.58–6.39)	.*281*	1.56 (0.31–8.00)	.*592*
Unknown	50%	3.48 (0.21–57.97)	.*385*	4.04 (0.20–80.08)	.*360*
PHQ‐2^j^	‐	‐	‐	‐	‐
Normal	23.0%	Ref.	‐	Ref.	‐
Abnormal	40.0%	2.23 (0.58–8.59)	.*243*	2.56 (0.54–12.14)	.*236*
Living alone	‐	‐	‐	‐	‐
No	23.3%	Ref.	‐	Ref.	‐
Yes	29.2%	1.36 (0.49–3.74)	.*553*	1.66 (0.47–5.93)	.*435*

Abbreviations: 6‐CIT, Six‐Item Cognitive Impairment Test; ADL, activities of daily living; CCI, Charlson comorbidity index; G8, geriatric 8; IADL, Instrumental Activities of Daily Living; MNA‐SF, Mini Nutritional Assessment—Short Form; PHQ‐2, patient health questionnaire‐2.

^a^ Multivariable logistic regression models were adjusted for age, sex, treatment intent, and tumor type. Sex, treatment intent, and tumor type were added to the model as binary variables and age was added as a continuous variable.

^b^ Frailty was defined as an impairment in ≥2 of 4 geriatric domains.

^c^ Defined as abnormal when G8 score ≤14.

^d^ Defined as CCI ≥1.

^e^ Defined as 5 or more medicines.

^f^ At risk of malnutrition when the score is ≤11.

^g^ Defined as dependent when ≤2.

^h^ Defined as dependent when ≤4 for men and ≤7 for women.

^i^ Defined as abnormal when >7.

^j^ Defined as abnormal when ≥3.

Among the impaired geriatric domains, patients with 3 or 4 impaired domains had a significantly higher risk of hospitalization compared to those with 0 or 1 impaired domains (OR: 8.85, 95%C.I.: 1.91–41.00, *p* = .005). Supplementary Figure 1b illustrates the significant hospital admission rates among palliative patients as the number of impaired domains increases (0 or 1, 2, and 3 or 4 impaired domains).

Multimorbidity showed a significant association with hospital admission risk only in the univariable analysis. Patients with a CCI score of ≥3 had an evident increased risk (OR: 5.54, 95%C.I.: 1.81–16.99, *p* = .003), whereas lower CCI scores showed weaker or non‐significant associations (CCI Score 1: OR: 1.16, 95%C.I.: 0.33–4.06, *p* = .816; CCI Score 2: OR: 1.14, 95%C.I.: 0.28–4.61, *p* = .015). Similarly, iADL impairment was significantly associated with hospital admission in the univariable analysis (OR: 2.67, 95%C.I.: 1.02–6.97, *p* = .045), but not in the multivariable analysis (OR: 2.87, 95%C.I.: 0.92–8.96, *p* = .069). Other GA components showed no significant association with the risk of hospitalization.

### 3.5 Predictive value of frailty, impaired GA components, and number of impaired domains for mortality

Frailty was significantly associated with increased all‐cause mortality risk (HR: 5.23, 95% C.I.: 1.81–15.11, *p* = .002). Supplementary Table 5 presents the causes of mortality among patients undergoing anti‐PD‐1 immunotherapy, stratified by frailty status. The majority of deaths (69%) were attributed to disease progression, making it the leading cause of mortality in both frail and non‐frail patients.

An increased number of impaired geriatric domains also predicted all‐cause mortality, with patients having 3 or 4 impaired domains showing a significantly higher risk of death compared to those with 0 or 1 impaired domains (HR: 15.04, 95% C.I.: 3.95–57.22, *p* <.001) in the multivariable analysis.

Several baseline GA components were associated with all‐cause mortality, including risk of malnutrition, impaired iADL, and abnormal scores on the PHQ‐2, indicating depressive symptoms (Table 4). While impaired G8, a CCI score of ≥3, and an impaired 6‐CIT showed associations with all‐cause mortality in univariable analyses, these associations were not significant in the multivariable models. Additionally, polypharmacy and falls in the last 6 months were not found to be significantly associated with all‐cause mortality.

**TABLE 4:** Predictive value of frailty, impaired geriatric domains and baseline GA components for mortality.

	% of 1‐year mortality within the subgroup	Univariable Cox regression Hazard ratio (95%C.I.)	*p*‐value	Multivariable Cox regression^a^ Hazard ratio (95%C.I.)	*p*‐value
Frailty^b^	‐	‐	‐	‐	‐
No	12%	Ref.	‐	Ref.	‐
Yes	36%	3.94 (1.72–9.02)	*<.001*	5.23 (1.81–15.11)	.*002*
Impaired geriatric domains	‐	‐	‐	‐	‐
0–1 (not frail)	12%	Ref.	‐	Ref.	‐
2 (frail)	23%	2.32 (0.92–5.86)	.*076*	3.90 (1.27–12.04)	.*018*
3–4 (very frail)	54%	9.42 (3.66–24.21)	*<.001*	15.04 (3.95–57.22)	*<.001*
Baseline GA components	‐				
G8 questionnaire^c^	‐	‐	‐	‐	‐
Normal	16%	Ref.	‐	Ref.	‐
Impaired	36%	2.64 (1.28–5.42)	.*008*	1.49 (0.63–3.52)	.*365*
CCI comorbidity^d^	‐	‐	‐	‐	‐
0	17%	Ref.	‐	Ref.	‐
1	23%	1.77 (0.64–4.90)	.*268*	1.22 (0.41–3.65)	.*723*
2	34%	2.17 (0.73–6.49)	.*164*	1.48 (0.44–4.98)	.*523*
≥3	35%	2.99 (1.12–7.93)	.*028*	1.69 (0.56–5.06)	.*351*
Polypharmacy^e^	‐	‐	‐	‐	‐
No	22%	Ref.	‐	Ref.	‐
Yes	31%	1.64 (0.84–3.22)	.*149*	0.82 (0.36–1.89)	.*641*
MNA‐SF^f^	‐	‐	‐	‐	‐
Well nourished	16%	Ref.	‐	Ref.	‐
Risk of malnutrition	35%	2.98 (1.46–6.09)	.*003*	2.52 (1.18–5.36)	.*017*
Falls in last 6 Months	‐	‐	‐	‐	‐
No	25%	Ref.	‐	Ref.	‐
Yes	32%	1.35 (0.61–2.98)	.*455*	1.63 (0.70–3.80)	.*257*
ADL^g^	‐	‐	‐	‐	‐
Independent	27%	Ref.	‐	Ref.	‐
Dependent	0%	NA^k^	*NA* ^k^	NA^k^	*NA* ^k^
IADL^h^	‐	‐	‐	‐	‐
Independent	23%	Ref.	‐	Ref.	‐
Dependent	35%	2.45 (1.23–4.91)	.*011*	2.43 (1.11–5.34)	.*027*
6‐CIT^i^	‐	‐	‐	‐	‐
Normal	24%	Ref.	‐	Ref.	‐
Impaired	44%	2.35 (1.06–5.24)	.*036*	1.01 (0.33–3.09)	.*984*
Unknown	0%	1.32 (0.18–9.88)	.*784*	1.95 (0.24–15.89)	.*534*
PHQ‐2^j^	‐	‐	‐	‐	‐
Normal	26%	Ref.	‐	Ref.	‐
Abnormal	32%	1.78 (0.69–4.62)	.*233*	3.09 (1.08–8.88)	.*036*
Living alone	‐	‐	‐	‐	‐
No	21%	Ref.	‐	Ref.	‐
Yes	44%	1.85 (0.91–3.78)	.*090*	1.51 (0.68–3.39)	.*315*

Abbreviations: 6‐CIT, Six‐Item Cognitive Impairment Test; ADL, activities of daily living; CCI, Charlson comorbidity index; G8, geriatric 8; IADL, Instrumental Activities of Daily Living; MNA‐SF, Mini Nutritional Assessment—Short Form; PHQ‐2, Patient Health Questionnaire‐2.

^a^ Multivariable Cox regression models were adjusted for age, sex, treatment intent, and tumor type. Sex, treatment intent, and tumor type were added to the model as binary variables, and age was added as a continuous variable.

^b^ Frailty was defined as an impairment in ≥2 of 4 geriatric domains.

^c^ Defined as abnormal when G8 score ≤14.

^d^ Defined as CCI ≥1.

^e^ Defined as 5 or more medicines.

^f^ At risk of malnutrition when the score is ≤11.

^g^ Defined as dependent when ≤2.

^h^ Defined as dependent when ≤4 for men and ≤7 for women.

^i^ Defined as abnormal when >7.

^j^ Defined as abnormal when ≥3.

^k^ An analysis was not performed due to an insufficient number of events recorded in this specific subgroup.

### 3.6 Clinical benefit from ICI in non‐frail versus frail patients treated in a palliative setting

Supplementary Figures 2a and 2b visually depict the proportions of patients who had clinical benefit from ICI, stratified by frailty status and categorized by the number of impaired geriatric domains.

## 4 DISCUSSION

This study showed that there was no association between geriatric impairments and the occurrence of grade ≥3 irAEs. However, frailty and the number of impaired geriatric domains were significantly associated with higher risks of all‐cause hospital admission and mortality. Only one component of the GA, multimorbidity (CCI score ≥3), showed a significant association with hospitalization. However, several baseline GA components were associated with all‐cause mortality, including the risk of malnutrition, impaired iADL, and abnormal scores on the PHQ‐2. Geriatric impairments were not associated with clinical benefit from ICI in patients treated with palliative intent.

The incidence of grade ≥3 irAEs in our cohort was consistent with previously published prospective studies, where similar rates were reported.^34^ Our findings did not show a significant difference in the incidence of grade ≥3 irAEs between fit and frail older patients, aligning with previous studies that categorized patients based on the G8 screening tool alone.^34^, ^35^ Even patients with deficits in 3 or more geriatric domains had no increased risk of grade ≥3 irAEs, reinforcing the robustness of our findings.

Previous studies found that frail older patients, identified by the G8 questionnaire, were more likely to experience hospital admissions due to irAEs or other causes, often with longer hospital stays.^34^, ^35^ While an impaired G8 score was associated with a higher frequency of hospitalization in our cohort, this finding was not statistically significant. When examining the reasons for hospital admissions in frail patients during anti‐PD‐1 immunotherapy treatment in our cohort, most were not directly related to immunotherapy, consistent with findings from a prior prospective study^35^ These findings highlight that in older frail patients undergoing immunotherapy, hospitalizations are often driven by pre‐existing vulnerabilities and non‐immunotherapy‐related issues rather than immune‐related toxicities alone. Therefore, all‐cause hospitalization is a clinically meaningful endpoint in this population, as it reflects the cumulative impact of treatment burden, comorbid burden, and reduced physiological reserve.

The lack of association between frailty and clinical benefit from ICIs in palliative care is particularly noteworthy. This finding suggests that frailty, while influencing treatment outcomes, does not compromise the therapeutic efficacy of ICIs. These results support the growing consensus that ICIs are generally well‐tolerated in older frail patients, provided their overall health is carefully managed. Despite similar rates of grade ≥3 irAEs between frail and non‐frail patients, frail patients were hospitalized more frequently, a particularly undesirable outcome in a palliative care setting. Their reduced life expectancy, often influenced by malignancy and comorbidities, further complicates treatment decisions. However, most hospitalizations were not directly related to immunotherapy itself but were instead due to non‐immunotherapy‐related causes, including palliative care needs, comorbidities, or disease progression. This raises the question of whether these hospitalizations could realistically be prevented. Additionally, even lower‐grade irAEs (grade 1–2) can disproportionately impact frail older patients, underscoring the need for tailored management strategies, such as closer monitoring, proactive symptom management, and modified treatment protocols.^21^

Our findings also highlight differences in treatment discontinuation between frail and non‐frail patients. Frail patients were more likely to discontinue therapy due to declining health status, consistent with prior studies showing a trend toward higher discontinuation rates with increasing frailty, possibly also due to older frail patients not tolerating grade 1–2 irAEs well.^21^ However, the persistence of immunotherapy effects after discontinuation raises questions about the clinical significance of early cessation. The Safe Stop trial is currently investigating the safety of early discontinuation of anti‐PD‐1 therapy in patients with advanced melanoma who achieve a confirmed tumor response.^36^ While the study hypothesizes that early discontinuation is safe and may maintain durable responses while improving quality of life and reducing treatment‐related toxicity, results are not yet available. Nevertheless, this approach warrants consideration in frail older adults, as it could support a lower threshold for pausing or discontinuing treatment in this vulnerable population, effectively balancing therapeutic efficacy with treatment burden, particularly in a palliative setting.

### 4.1 Strengths and limitations

The TENT and IMAGINE studies are multicenter, prospective studies including substantial numbers of frail and non‐frail older patients, overcoming common challenges in frail patient recruitment due to chronic conditions and limited energy for study participation. To minimize patient burden, baseline geriatric assessments were mostly conducted during immunotherapy treatment or via telephone, and follow‐up assessments were carried out via telephone. However, the relatively small sample size in our study could be attributed to various factors, including challenges in patient enrollment, as participation in multiple studies was perceived as burdensome by both older patients and oncologists. Nevertheless, our cohort included a substantial proportion of frail older adults, allowing for insightful analyses within this population. In interpreting our findings, it is important to consider that comorbidities and tumor types may share common etiological factors, which could have impacted the observed associations in our study. For example, smoking is a risk factor for both chronic obstructive pulmonary disease and lung cancer. This overlap could have confounded the association between comorbidities and mortality in our study. Adjusting for tumor type might have absorbed some of the effect of comorbidities, potentially explaining why the association between comorbidity and mortality was significant in the univariable analysis but lost significance in the multivariable analysis. Subgroup analysis also yielded wide CIs due to the modest sample size. Furthermore, our study only included grade ≥3 irAE data as they typically require initiation of medication, making them more clearly identifiable in patient records; frail patients may also experience significant impacts from grade 1–2 irAEs.

In summary, frailty was not significantly associated with the occurrence of grade ≥3 irAEs. However, frailty and the number of impaired geriatric domains were significantly associated with higher risks of hospital admission and mortality in older frail patients receiving anti‐PD‐1 therapy, underscoring the importance of GA in frail older patients to guide treatment decisions and balance therapeutic efficacy with treatment and disease burden, particularly in the palliative setting. Hospitalizations in older frail patients were often driven by pre‐existing vulnerabilities and non‐immunotherapy‐related issues rather than immune‐related toxicities. Therefore, frailty should not be considered a contraindication for ICI therapy, as this therapy is generally well‐tolerated, even among older frail patients. Instead, frailty should be viewed as a relevant clinical factor for optimizing therapeutic decision‐making and tailoring supportive interventions in older patients.

These findings highlight the need for further research to determine whether interventions guided by geriatric assessments can reduce hospital admissions and investigate critical patient outcomes, such as quality of life. Such efforts are essential to refine treatment approaches and enhance supportive care for this vulnerable population.

## AUTHOR CONTRIBUTIONS

**Asli Özkan:** Conceptualization; methodology; data curation; visualization; formal analysis; project administration; writing – original draft. **Karlijn de Joode:** Conceptualization; data curation; project administration; writing – review and editing. **Ellen Kapiteijn:** Data curation; writing – review and editing. **Marije Slingerland:** Data curation; writing – review and editing; project administration. **Stephanie Zunder:** Data curation; writing – review and editing. **Frederiek van den Bos:** Conceptualization; methodology; data curation; writing – review and editing. **Simon Mooijaart:** Writing – review and editing. **Anna Uit den Boogaard:** Data curation; writing – review and editing. **Stella Trompet:** Methodology; conceptualization; data curation; writing – review and editing. **Hans Westgeest:** Data curation; writing – review and editing. **Astrid van der Veldt:** Conceptualization; writing – review and editing; data curation. **Ron H. J. Mathijssen:** Conceptualization; data curation; writing – review and editing. **Geert Labots:** Data curation. **Cynthia Holterhues:** Data curation. **Els M. E. Verdegaal:** Writing – review and editing. **Nienke A. de Glas:** Conceptualization; methodology; data curation; supervision; funding acquisition; project administration; writing – review and editing. **Johanneke E. A. Portielje:** Conceptualization; methodology; data curation; supervision; writing – review and editing.

## FUNDING INFORMATION

This study was supported by a personal grant awarded to N. A. de Glas by the Leiden University Medical Center and the ZonMW/Veni program (09150161810003). This work was supported by Stichting Fonds Oncologie Holland (project number 22‐01) and Leiden University Fund project (W20361‐2‐38). The funding organizations had no role in the study design, data collection, analysis, or interpretation of data, the writing of the manuscript, or the decision to submit the manuscript for publication.

## CONFLICT OF INTEREST STATEMENT

K. de Joode declares travel costs from Ipsen, not related to the current work. H. Westgeest has received honoraria from Merck (2022). E. Kapiteijn has consultancy/advisory relationships with Delcath, Immunocore, and Lilly, and received research grants not related to this paper from Bristol Myers Squibb, Delcath, Novartis, and Pierre‐Fabre. Not related to current work and paid to the institute. R. H.J. Mathijssen received research grants not related to this paper and paid to the institute from Astellas, Bayer, Boehringer‐Ingelheim, Cristal Therapeutics, Deuter Oncology, Nordic Pharma, Novartis, Pamgene, Pfizer, Roche, Sanofi, and Servier. A. van der Veldt received consultancy fees (all paid to the institute) from: BMS, MSD, Ipsen, Novartis, Sanofi, Pfizer, Eisai, Roche, Pierre Fabre; travel costs from Ipsen. No potential conflict of interest was disclosed by the other authors. The funders had no role in the design of the study; in the collection, analyses, or interpretation of data; in the writing of the manuscript, or in the decision to publish the results.

## ETHICS STATEMENT

The study protocols for the TENT and IMAGINE studies were reviewed and approved by the Medical Ethics Committee of Leiden The Hague Delft. The TENT study was registered with the Central Committee on Research Involving Human Subjects (CCMO) under approval number NL53575.058.15, and the IMAGINE study was registered under approval number NL59959.058.17. Written informed consent was obtained from all patients in accordance with the Declaration of Helsinki.

## Supporting information


**Data S1:** Supporting Information

## Data Availability

The data that support the findings of this study are available from the corresponding author upon reasonable request.
