# Supplementary material for: Impact of geriatric impairments on outcomes of single‐agent immunotherapy in solid tumors
Source: Int J Cancer. 2025 Oct 4;158(5):1370–82. doi: 10.1002/ijc.70185 (PMC12765961; doi:10.1002/ijc.70185)
Supplement: Supplementary file 1 — Data S1: Supporting Information [file IJC-158-1370-s001.pdf]

Impact of Geriatric Impairments on Outcomes of Single-Agent Immunotherapy in Solid Tumors

Asli Özkan, Karlijn de Joode, Ellen Kapiteijn, Marije Slingerland, Stephanie Zunder, Frederiek van den Bos, Simon Mooijaart, Anna Uit den Boogaard, Stella Trompet, Hans Westgeest, Astrid van der Veldt, Ron H. J. Mathijssen, Geert Labots, Cynthia Holterhues, Els M. E. Verdegaal, Nienke A. de Glas, Johanneke E. A. Portielje.

Table of contents

Supplementary Tables

- Supplementary Table 1. Overview of the Thresholds for Geriatric Impairment Within Assessment/Screening Tools Used Across Domains in the Included Studies.
- Supplementary Table 2. Summary of Grade ≥3 Immune-Related Adverse Event During Anti-PD-1 Immunotherapy Treatment
- Supplementary Table 3. Overview of Reasons for Early Discontinuation of ICI
- Supplementary Table 4. Overview of Reasons for Hospital Admission During Anti-PD-1 Immunotherapy Treatment
- Supplementary Table 5. Causes of Mortality During Anti-PD-1 Immunotherapy Treatment

Supplementary Figures

- Supplementary Figure 1a. Hospital Admission in Palliative Patients at 12 Months, Stratified by Frailty Status
- Supplementary Figure 1b. Hospital Admission in Palliative Patients at 12 Months, Stratified by the Number of Impaired Domains (0 or 1, 2, and 3 or 4 Domains)
- Supplementary Figure 2a. Clinical Benefit (CR, PR or SD) from ICI at 6 Months in Fit Versus Frail Patients Receiving Treatment in a Palliative Setting
- Supplementary Figure 2b. Clinical Benefit from ICI at 6 Months in Palliative Patients, Stratified by the Number of Impaired Domains (0 or 1, 2, and 3 or 4 Domains)

Supplementary Table 1. *Overview of the Thresholds for Geriatric Impairment Within Assessment/Screening Tools Used Across Domains in the Included Studies.*

Domain*	Geriatric Assessment/Screening Tool (threshold)**
Somatic	• Charlson Comorbidity Index (≥1)***<br>• Polypharmacy (≥5)<br>• Mini Nutritional Assessment Short-Form (≤11)
Functional	• Falls ≤6 months<br>• Institutionalization<br>• Functional dependency<br>o KATZ-ADL (≥2)<br>o LAWTON-IADL (Men ≤4, Women ≤7)
Mental	• Dementia<br>• Six-Item Cognitive Impairment Test (>7)<br>• Patient Health Questionnaire-2 ≥3
Social	• Considered abnormal if patient lived alone

** Patients who scored abnormally on ≥ 2 of 4 geriatric domains were classified as frail.*

*** If ≥1 tests in the somatic, functional, mentall, and/or social domains were scored abnormal, the domain was considered abnormal.*

**** Comorbidities were assessed by the Charlson Comorbidity index (CCI). No points were assigned to the 'solid tumor' category if no other tumors were present as this was considered to be the main diagnosis. Therefore, presence of comorbidity was defined as CCI ≥1.*

Supplementary Table 2. Summary of Grade ≥3 Immune-Related Adverse Event During Anti-PD-1 Immunotherapy Treatment

	Total N=19	*%*
Pneumonitis	4	21.1
Colitis	3	15.8
Thyroid dysfunction	3	15.8
Hepatitis	2	10.5
Adrenal insufficiency	1	5.3
Nephritis	1	5.3
Other	5	26.3

Supplementary Table 3. Overview of Reasons for Early Discontinuation of ICI

	Total N=47	*%*	*Frail patients (N=33)*	*Non-frail patients (N=14)*
Progression	23	48.9	17	6
irAEs	20	42.6	12	8
Poor patient condition	4	8.5	4	0

Supplementary Table 4. Overview of Reasons for Hospital Admission During Anti-PD-1 Immunotherapy Treatment

	Total N=27	*%*	*Frail patients (N=21)*	*Non-frail patients (N=6)*
Immunotherapy-related adverse event	7	25.9	5	2
Non-Immunotherapy-Related Cause				
Palliative care	6	22.2	4	2
Disease progression	3	11.1	3	0
Infectious disease (e.g., pneumonia)	2	7.4	1	1
Pulmonary embolism/ Deep vein thrombosis	2	7.4	2	0
Hemoptysis	2	7.4	2	0
Gastrointestinal bleeding	1	3.7	0	1
Hyperglycemia	1	3.7	1	0
Collaps	1	3.7	1	0
Unspecified non-immunotherapy related admissions	2	7.4	2	0

Supplementary Table 5. Causes of Mortality During Anti-PD-1 Immunotherapy Treatment

	Total N=29	*%*	*Frail patients (N=23)*	*Non-frail patients (N=6)*
Disease Progression	20	69.0	16	4
Comorbidity	4	13.8	3	1
Unknown	5	17.2	4	1

Supplementary Figure 1a. Hospital Admission in Palliative Patients at 12 Months, Stratified by Frailty Status

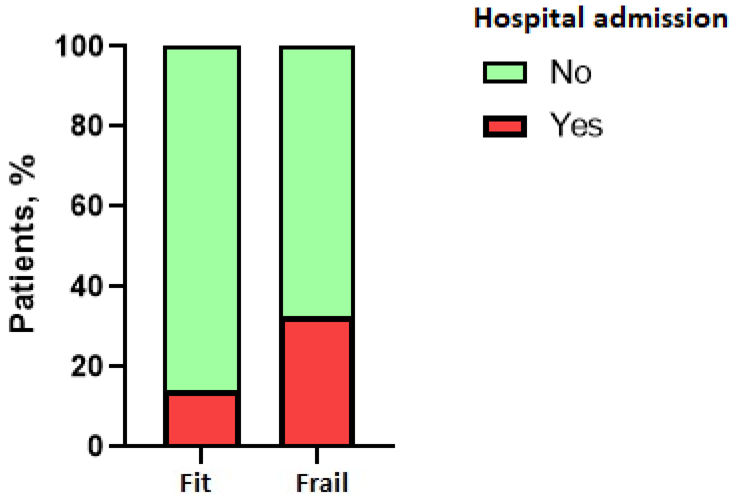

OR: 3.04, 95%C.I.: 0.91-10.17, p=0.070.

Supplementary Figure 1b. Hospital Admission in Palliative Patients at 12 Months, Stratified by the Number of Impaired Domains (0 or 1, 2, and 3 or 4 Domains)

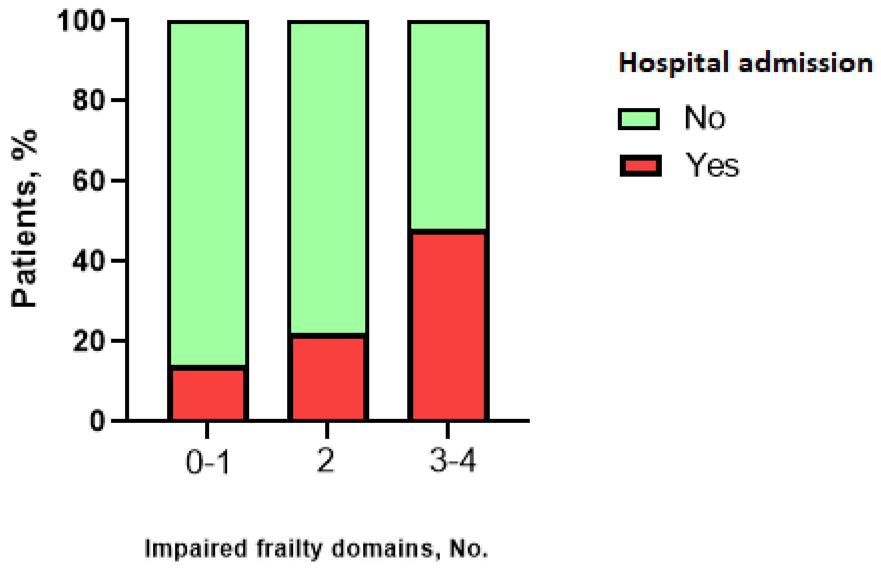

0-1 domain (Ref.), 2 impaired domains; OR: 1.75 (95%C.I.: 0.45-6.80) p=0.419, 3-4 impaired domains; OR 5.75 (95%C.I.: 1.49-22.21) p=0.011.

Supplementary Figure 2a. Clinical Benefit (CR, PR or SD) from ICI at 6 Months in Fit Versus Frail Patients Receiving Treatment in a Palliative Setting

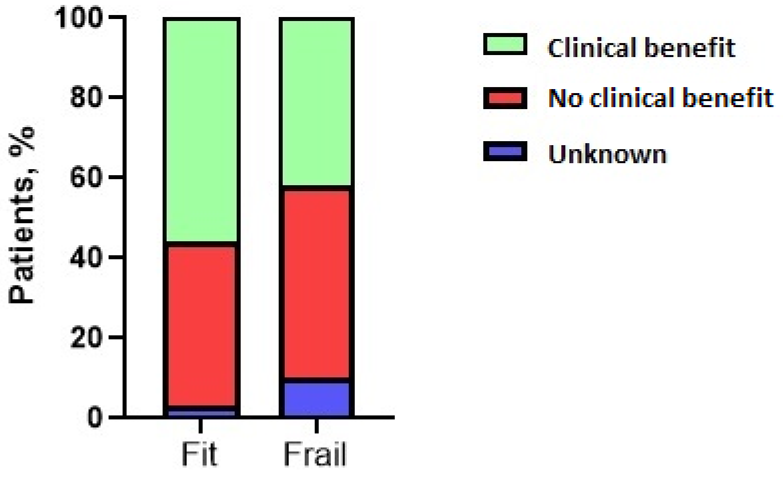

OR: 1.55, 95%C.I.: 0.59-4.07, p=0.374.

Supplementary Figure 2b. Clinical Benefit from ICI at 6 Months in Palliative Patients, Stratified by the Number of Impaired Domains (0 or 1, 2, and 3 or 4 Domains)

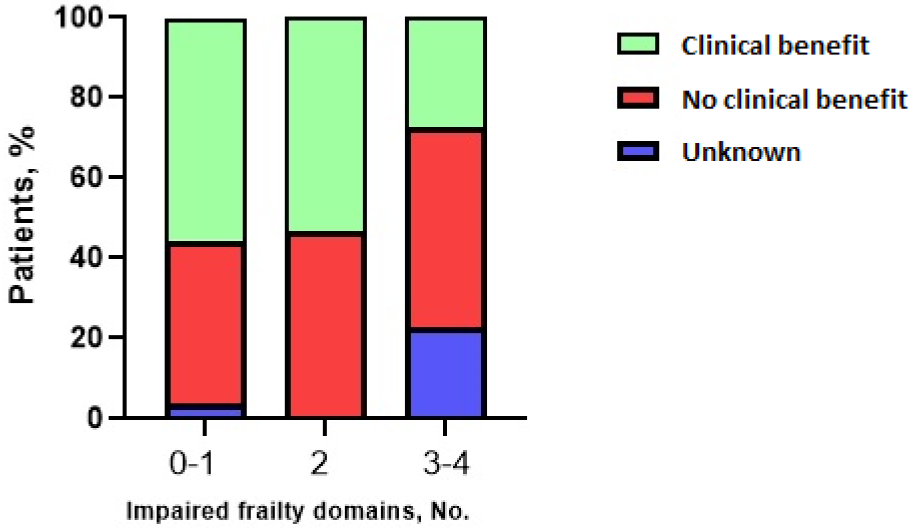

0-1 domain (Ref.), 2 impaired domains; OR: 1.19 (95%C.I.: 0.41-3.44) p=0.744, 3-4 impaired domains; OR 2.50 (95%C.I.: 0.71-8.84) p=0.155.
